# Emergent Systems Energy Laws for Predicting Myosin Ensemble Processivity

**DOI:** 10.1371/journal.pcbi.1004177

**Published:** 2015-04-17

**Authors:** Paul Egan, Jeffrey Moore, Christian Schunn, Jonathan Cagan, Philip LeDuc

**Affiliations:** 1 Department of Mechanical Engineering, Carnegie Mellon University, Pittsburgh, Pennsylvania, United States of America; 2 Department of Physiology and Biophysics, Boston University School of Medicine, Boston, Massachusetts, United States of America; 3 Department of Psychology, University of Pittsburgh, Pittsburgh, Pennsylvania, United States of America; Yale University, UNITED STATES

## Abstract

In complex systems with stochastic components, systems laws often emerge that describe higher level behavior regardless of lower level component configurations. In this paper, emergent laws for describing mechanochemical systems are investigated for processive myosin-actin motility systems. On the basis of prior experimental evidence that longer processive lifetimes are enabled by larger myosin ensembles, it is hypothesized that emergent scaling laws could coincide with myosin-actin contact probability or system energy consumption. Because processivity is difficult to predict analytically and measure experimentally, agent-based computational techniques are developed to simulate processive myosin ensembles and produce novel processive lifetime measurements. It is demonstrated that only systems energy relationships hold regardless of isoform configurations or ensemble size, and a unified expression for predicting processive lifetime is revealed. The finding of such laws provides insight for how patterns emerge in stochastic mechanochemical systems, while also informing understanding and engineering of complex biological systems.

## Author Summary

Complex biological systems consist of many parts that interact in non-obvious ways. In these systems, levels of organization often emerge, as evidenced by cases where cells form tissues, tissues form organs, and organs interact to form complete organisms. We hypothesized that that laws exist that describe system functioning at one level, independently of the configuration at other levels. The hypothesis was tested using simulations of motor protein systems, and demonstrated that patterns in their behavior emerge at a systems level. Results demonstrated a law concerning energy utilization predicts the lifetime of these systems before dissociation, regardless of the components present in the system. These findings reveal organizational laws that simplify complex systems analysis and can facilitate engineering design approaches for bio-based technologies.

## Introduction

In multi-level stochastic systems, the collective interactions of lower-level building blocks are necessary for producing emergent system functionality, however, some emergent system properties may hold regardless of how lower level building blocks are configured [1]. This general principal is highly applicable to biophysical systems, where complex system functionality emerges from stochastic mechanochemical molecular interactions [2–4]. Collective emergent functionality is a definitive feature of motility systems, where filament gliding behavior emerges from the interactions of myosin molecular motors and actin filaments [5,6]. To promote motility, myosins exert force as they stochastically attach and detach to gliding actin filaments [6–8]. However, it is not fully understood how changes in myosin isoform structure affect the system’s higher level functioning (e.g. how fast/long the filament continues gliding). Such considerations are important because many isoforms exist in the myosin superfamily, with particular isoforms suited for varied cellular functions including muscular contraction, cytoskeleton scaffolding, and active diffusion [9]. Better descriptions for how individual myosin structure [10] and ensemble size [11] relate to system functionality could promote understanding of both natural and engineered molecular motor systems [12,13]. Derived system laws that describe the operations at the systems level as components are altered could significantly advance analyses of natural and synthetic myosin performance [14,15], and have particular applications relating to myosin-based diseases such as cardiomyopathy, where muscle tissue growth is affected by individual myosin configuration [16]. Additionally, such rules could aid in developing heuristics for engineered technologies such as nano-actuators, molecular materials, and bio-sensors [17,18].

**Fig 1:**
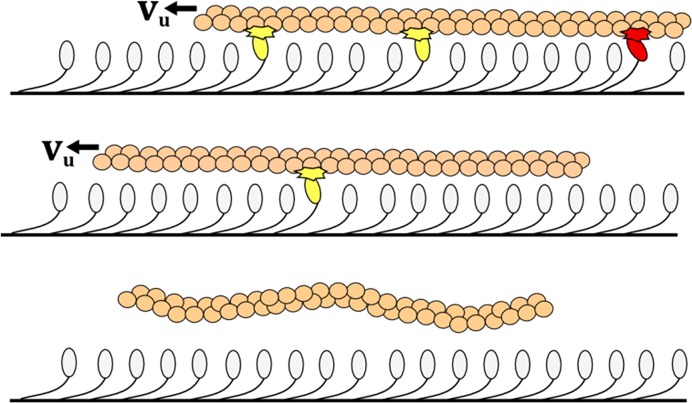
Schematic of processive myosin system with dissociation. Schematic of a myosin ensemble propelling actin at unloaded velocity *v*_*u*_. Myosin states are stochastic, with myosins being detached, attached and power-stroking (light yellow point of contact), or attached and drag-stroking (dark red point of contact). Initially three myosins are attached (top); later the filament has translated and one myosin is attached (middle); at processivity termination, all myosins are detached (bottom).

In both natural and engineered myosin systems, functionality often emerges from the processive transport of actin filaments relative to stationary myosins; a minimum number of myosins are required to ensure a filament continues with a consistent trajectory and velocity. Consistency in processivity is measurable through considering a system’s processive lifetime P, which refers to the duration from initial myosin-actin contact until system dissociation occurs during periods when no myosins are in contact with actin (Fig 1) [19]. Processivity is an essential metric to consider in the design of myosin-based nanotechnologies that operate on similar principles as motility assays [20]. Motility assays are common experiments for investigating how individual myosin configuration affects system behavior, and these experiments often measure the velocity of actin filaments propelled by a bed of myosins [21]. Typically, there is a negligible load assumed to act on the actin filament, which is representative of physiological situations with low external loads or nanotechnologies that operate in similar controlled environments. Although many models and simulations exist for myosin systems [5,22–24], they mostly concentrate on physiological models rather than motility assays. The simulation of motility assays, however, enables the experimental investigation of phenomenon such as how myosin isoform configuration affects the maximum achievable filament velocity and probability of contact among myosins and actin, which is suggestive of the potential loaded system capabilities. We therefore concentrate on building models and simulations of motility assays as a basis for investigating emergent system laws informed by fundamental biophysical experiments.

From previous motility assay experiments, P is known to increase with the number of myosins *N* interacting with a filament and myosin duty ratio *r*, defined as the proportion of time a myosin remains attached to actin [25]. Because processivity is a system behavior that emerges from many myosins’ stochastic actions [26], none of which are processive individually, it is not possible to predict P with certainty, but rather requires the consideration of an average. These complications have made quantifications of P difficult and established analytical models have instead relied on estimations [19,27] to predict whether a system behaves in an emergent regime [28] that enables perpetual processivity. These estimations require consideration of the contact probability of a system *P*_*C*_ (the probability of at least one myosin being attached at a given time), as

$$P_{c}=1-{\left(1-r\right)}^{N}$$ (1)

Experimental studies suggest that perpetual processivity is reached for chicken skeletal muscle myosin when *P*_*C*_ ≥ 90% [19], but could require *P*_*C*_ ≥ 99.6% [29] when considering uncertainties. These uncertainties arise from standard error in average filament velocity measurements at the systems level, and in determining specific behaviors of individual myosins, such as their rate of attachment which is not directly measureable. Because of these uncertainties and their propagation across scales, it is difficult to determine with precision the influence of particular parameters such as *P*_*C*_ on system performance, since it is influenced by multiple measurements with uncertainty. Therefore, simulation approaches informed by empirical measurements can enable insights for how difficult to measure parameters affect system behavior.

It is not obvious whether processive lifetime P would scale with contact probability *P*_*C*_ as myosin isoform structure varies and influences duty ratio *r* (i.e. will systems with equivalent *P*_*C*_ have similar processive lifetimes), which is representative of an initial hypothesis. As an alternative, we propose that system energy consumption *E* increases with the number of myosins in a system/ensemble *N*, which assumes each myosin continues utilizing ATP at the same rate as more myosins are added to the system, and generally holds true for unloaded systems. It can then be hypothesized that such energy relationships could inform a scaling law, since it would support previous empirical measurements that processive lifetime increases as myosins are added to the system. With energy scaling, it is assumed that myosins consume energy from ATP at a rate *e*, and *E* = *e*∙*N*, which is a valid assumption if *N* does not influence e. Generally this assumption is true when adding more myosins to the system does not affect the individual force output of myosins. The cycling rate of myosins is primarily governed by the velocity of the gliding filament, and eventually saturates at higher velocities [27], meaning that the linear assumption is valid for systems operating at high velocities. Such scenarios are possible when the force on the system is low, such as muscle contraction against low loads or in vitro motility studies where filaments must only overcome the drag force from their environment; in both cases, adding or removing myosins from the system does not significantly alter the gliding filament velocity. It is then possible to assume the energy required for perpetual processivity by approximating that *P*_*C*_ ≈ 90% when an average number of attached motors is *N*_*att*_ = *N* ∙ *r* ≈ 2; a minimum required *N* for perpetual processivity occurs when *N* = 2/*r*, or *E* = 2 ∙ *e/r* if P scales with system energy.

In this paper, our goal is to determine how P scales when considering *P*_*C*_ and *E*, and then develop a unified expression for predicting P regardless of isoform configuration and *N*. These predictions are possible to validate with wet-lab experiments, but infeasible to conduct for every possible system configuration. Where empirical data is not available, analytical approaches are utilized to validate the predictions of the simulation through predicting the system behavior of the same system configuration input in both models. The unified expression would greatly aid in predicting mechanisms of complex *in vivo* biological systems while enabling rapid prototyping of myosin-based technologies [12]. The isoform parameters chosen in this paper are the myosin lever arm length, myosin detachment rate, and myosin attachment rate which are three of the most critical parameters for optimizing myosins for nanotechnologies[18]. These parameters roughly correspond to the three primary phases of a myosin’s cycle consisting of a power-stroke (positive force generation), drag-stroke/detachment (negative force generation), and velocity dependent rate of binding to actin (Fig 1) [15]. All three of these parameters have values investigated empirically for a number of different isoforms and their influence on motility behavior, which aids in validation of developed models and simulations.

Because of the intractability of predicting processive ensemble behavior with analytical models, the problem is approached using agent-based simulation methods [30,31], where each agent is an individually configured computational object that autonomously recreates myosin behaviors in a spatially discretized virtual environment. The approach contrasts to past myosin simulations focusing on muscle [5,24,32], which are challenging to validate for single myosin functioning. We have previously examined myosin force-velocity curves for varied isoforms and developed a discretized virtual motility assay environment [15]. Here, analytical expressions and simulations are first examined for varied isoform types and validated with experimental evidence of how altered myosin molecular structures affect ensemble behavior. A computational environment is then built for simulating and measuring processive lifetime P durations, and these novel measurements are used to determine whether P scales with contact probability *P*_*C*_ or system energy *E* modulation. Once a scaling metric is found that is independent of isoform configuration, an analytical expression is fit to the simulation data that serves as the unified expression for determining P of any system regardless of its components.

## Methods

### Analytical and Agent-Based Modeling Approaches

Analytical and simulation approaches were first developed with a three-state mechanochemical model for individual myosins interacting with an actin filament traveling at steady state velocity *v* without regards to processive lifetime P (Supplementary Movie 1 in S1 Text), that is an extension of past myosin modeling methods [27]. When a myosin is detached, it stores energy from ATP as strain while the myosin head is displaced from its equilibrium state and attaches to actin sites with attachment rate *k*_*on*_. Binding sites are spaced every *x*_*d*_ (36nm) distance, based on actin’s highly conserved structure. Once attached, the myosin has displacement δ_+_ in its power-stroke, that decreases before reaching a point of zero-displacement based on lever length *l* and step angle *θ*, with *δ*_+_ = *l* ∙ *sin*(*θ*), with *θ* assumed as 30° for all isoforms considered in this study. Once a myosin reaches zero-displacement, it begins displacing in a negative direction as other myosins continue pulling the filament. The myosin detaches with rate constant *k*_*off*_ during its drag-stroke of length *δ*_−_ = *v/k*_*off*_. *δ*_+_, *k*_*on*_, and *k*_*off*_ were determined as isoform parameters because they map to molecular structures that vary independently in nature [33,34] and are prevalent in myosin engineering experiments [14,35–37].

Because myosins behave stochastically, it is often not possible to predict with certainty a myosin’s state and therefore time-average behaviors are typically considered. Time-average behaviors are validated from analytical and simulation predictions with experimental evidence from past unloaded motility assays, for varied isoforms. In unloaded assays, myosins propel the filament at unloaded velocity *v*_*u*_ and forces produced by power-stroking myosins balance drag-strokers, such that the time-average myosin displacement 〈*d*〉 equals zero. 〈*d*〉 is found by considering myosins with complete stroke lengths *δ*_*on*_ = *δ*_+_ + *δ*_−_, leading to 〈*d*〉 = (*δ*_+_/2) ∙ (*δ*_+_/*δ*_*on*_) – (*δ*_−_) ∙ (*δ*_−_/*δ*_*on*_) [27]. When 〈*d*〉 = 0, $\delta_{+}^{2}/2=\delta_{-}^{2}$, and simplifies to $\delta_{+}^{2}/2={\left(v_{u}/k_{off}\right)}^{2}$, to produce

$$v_{u}=\left(\sqrt{2}/2)∙(\delta_{+}∙k_{off}\right)$$ (2)

suggesting that *v*_*u*_ is linearly dependent on *δ*_+_ and *k*_*off*_. In cases when motility systems operate under load, the time-average force is calculated by 〈*f*〉 = *κ* ∙ *r* ∙ 〈*d*〉, where the myosin stiffness *κ* is found through considering that a myosin can not store energy greater than what myosins may approximately utilize from ATP (*e* = 62.5*zJ*) to find *κ* = *e*/*δ*_+_^2^.

A myosin’s duty ratio *r* is found analytically by modifying past methods [27] and assuming a filament has traveled *x* distance at time *t*, with a myosin head having probability *p*_*on*_(*x*,*t*) and *p*_*off*_(*x*,*t*) of attaching and detaching to binding sites, respectively. With rate constants for attaching *k*_*on*_(*x*) and detaching *k*_*off*_(*x*), a myosin’s interaction with actin when travelling at steady state is:

$$p_{on}\left(x\right)=\frac{v\frac{\partial p_{on}}{\partial x}\left(x\right)+k_{on}\left(x\right)p_{off}\left(x\right)}{k_{off}\left(x\right)}$$ (3)

When considering *k*_*on*_ as a high rate of attachment that occurs for a myosin head within a spatial proximity *x*_*z*_ to a binding site, and that myosins only bind while detached, the probability of binding *P*_*on*_ over time *t*_0_ as a site passes is:

$$P_{on}=\overset{t_{0}}{\underset{0}{\int }}k_{on}exp\left(-k_{on}t_{0}\right)dt=\;1-exp\left(-k_{on}t_{0}\right)=1-exp\left(\frac{-k_{on}x_{z}}{v}\right)$$ (4)

Because binding sites are spaced regularly by *x*_*d*_, the average distance *Δ* a filament travels each myosin cycle is

$$\Delta =\frac{x_{d}}{1-exp\left(-\frac{k_{on}∙x_{z}}{v}\right)}$$ (5)

and *r* = *δ*_*on*_/*Δ*.

**Fig 2:**
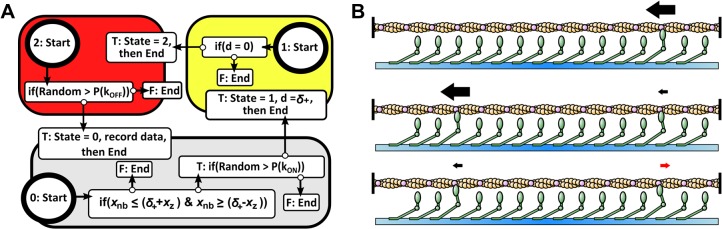
Agent-based simulation of myosin systems. (a) Logic rules that each individual myosin agent autonomously follows each step of the simulation. (b) Three rendered frames of myosin ensembles interacting with a single long actin filament. Myosins only generate force when attached to actin and based on their state generate positive force promoting filament motility (left pointing arrows) or negative force retarding motility (right pointing arrows).

The agent-based simulation consists of a discrete number of independently configured and autonomous myosin agents interacting with a filament in a virtual environment. The simulation operates in discrete spatial and temporal steps, with a filament translating *dX* = 1*nm* each step over a duration *dT* determined by *v* = *dX*/*dT*, which is a small enough step size to capture individual myosin behaviors while keeping required computational effort to a minimum. Each myosin agent has three possible states of either (0) detached, (1) being attached to the filament during a power-stroke, or (2) being attached to the filament during a drag-stroke. During each timestep of the simulation, each myosin agent follows programmed logic as presented Fig 2A that is representative of a myosin’s mechanochemical states and behaviors. Depending on a myosin’s current state, it will begin following rules in one of three ‘Start’ blocks and continue through if/then statements until an ‘End’ command is reached. For instance, a myosin that is not bound to actin (state 0) will first check if a binding site is near, where *x*_*nb*_ is the distance from a myosin’s zero strain location to the nearest binding site, *x*_*z*_ represents how close a myosin head must be to a binding site to have a chance of binding, and step size *δ*_+_ represents the distance of a myosin head from the point of zero strain. If the check fails, the myosin ceases its actions until the next time step. If the check succeeds, a random number is generated and compared to a myosin’s chance of attachment for that time step to determine whether it binds and enters the power-stroke state for the next simulation step or ceases its actions. The chance to attach is based on the actin's attachment rate parameter *k*_*on*_ and the window of time a binding site is available such that *P*(*k*_*on*_) = *k*_*on*_ ∙ *dT*. If a myosin agent attaches, it remains in its power-stroke (state 1) until it has a head displacement *d* of zero, as its head translates with the travelling filament and has initial displacement *d* = *δ*_+_ that reduces by *dX* each step. In the drag-stroke (state 2), a myosin has a random chance of detaching according to its detachment rate *k*_*off*_ and *P*(*k*_*off*_) = *k*_*off*_ ∙ *dT*. Fig 2B demonstrates a rendering of myosins operating as an ensemble according to the rules in Fig 2A.

In order to compare the simulation with analytical results, the time-average force 〈*f*〉 is required which represents the behaviors of myosin agents interacting with a filament travelling at *v*. The velocity of a simulation is assumed *a priori* and the time and spatial steps are then calculated. Monte Carlo samplings were used to determine a myosin’s displacement at time *t*, and 〈*f*〉 is found from aggregating *m* measurements such that $\langle f\rangle =\frac{1}{m}\sum_{i=1}^{m}f(t_{i})$, where *f*(*t*_*i*_) is a motor’s instantaneous force at time *t*_*i*_ found by *f* = *k* ∙ *d*. The simulation terminates once the standard error *s*_*e*_ of the mean for 〈*f*〉 reaches *s*_*e*_ ≤ 0.005, therefore *m* varies each simulation. In these simulations velocity is considered an independent variable while force is a dependent variable, therefore iterative processes in assuming velocities is required in cases where the simulations are utilized to determine the velocity of a system under a specified load. In the case of motility assays, the external load is considered to be zero and an initial assumption for velocity is found through using the analytical equations.

## Results

### Validation of Analytical Model and Agent-Based Simulation

**Fig 3:**
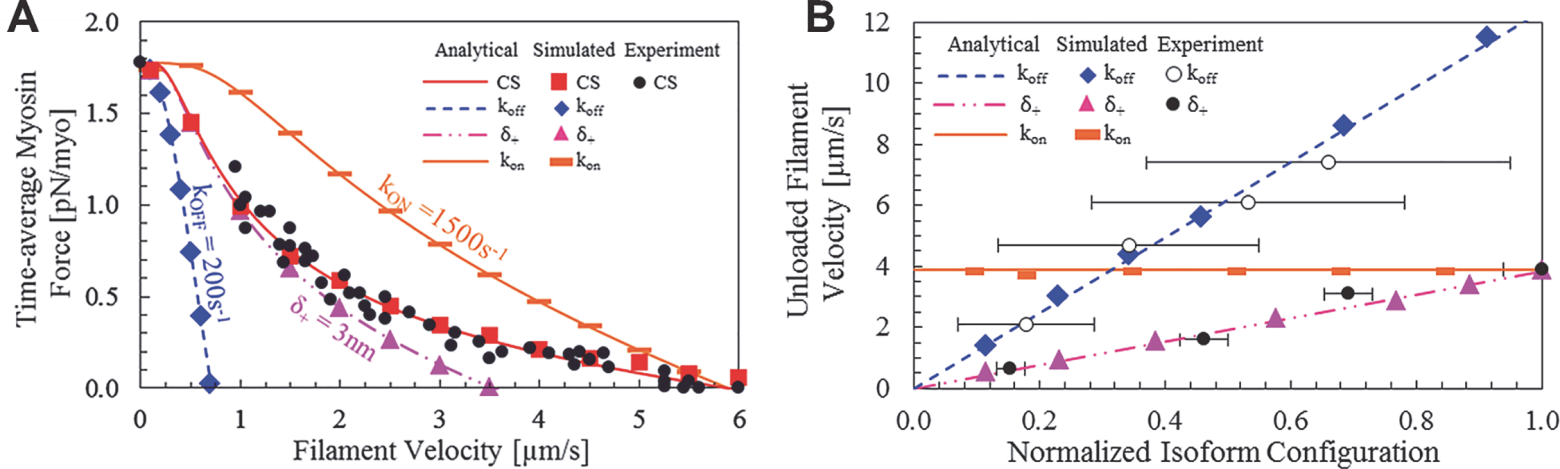
Comparison of agent-based molecular simulation and analytical methods to experimental data. (a) A datum isoform (squares) has *k*_*on*_ = 900*s* ^−1^, *k*_*off*_ = 1600*s* ^−1^, and *δ*_+_ = 5*nm* that correspond to empirical measurements [27], whereas extrapolated isoforms have one perturbed parameter each as labeled on the chart (e.g. the “*k*_*on*_ = 1500*s* ^−1^” isoform has values *k*_*on*_ = 1500*s* ^−1^, *k*_*off*_ = 1600*s* ^−1^, and *δ*_+_ = 5*nm*). Each line corresponds to analytical outputs while symbols refer to simulation data, with the exception of solid circles that represent experimental data. (b) *v*_*u*_ as myosin isoforms vary for analysis and simulations, with each isoform normalized to one perturbed parameter as other parameters remain constant. The *k*_*off*_ perturbation (blue diamonds) has *k*_*on*_ = 900*s* ^−1^, *k*_*off*_ = 3500*s* ^−1^, and *δ*_+_ = 10*nm*, normalization; the *k*_*on*_ perturbation (orange rectangles) has *k*_*on*_ = 3500*s* ^−1^, *k*_*off*_ = 1000*s* ^−1^, and *δ*_+_ = 10*nm* normalization; the *δ*_+_ perturbation (pink triangles) has *k*_*on*_ = 900*s* ^−1^, *k*_*off*_ = 800*s* ^−1^, and *δ*_+_ = 13*nm* normalization. Experimental data corresponds to the *δ*_+_ [14] and *k*_*off*_ [33] values.

The analytical and simulation models were validated by comparing empirical data [27] for chicken skeletal muscle myosin (*k*_*on*_ = 900*s* ^−1^, *k*_*off*_ = 1600*s* ^−1^, and *δ*_+_ = 5*nm*) under load to isoforms with one configuration variable altered, while the remaining two are identical to chicken skeletal myosin as indicated in Fig 3A. The force-velocity relationship was found analytically through solving 〈*f*〉 = *κ* ∙ *r* ∙ 〈*d*〉 as described further in the methods section, while the simulated force-velocity relationship was determined through simulating ensemble systems at varied velocities and aggregating to find the time-average force until error was negligible.

The resulting force velocity relationships demonstrate that both the analytical and simulation methods can recreate the hyperbolic force-velocity relationship of myosins, which forms the basis of muscle performance. Additionally, each isoform configuration parameter has unique influences on systems functioning, such as higher attachment rates leading to a greater force per velocity with no influence on maximum unloaded velocity, while step size and detachment rate decreases result in lower maximum velocities and force per velocity. These differences are important, because it suggests a complex relationship in emergent ensemble behavior based on individual myosin configuration. Notably, attachment rate increases result in greater energy expenditure in a system (because myosins cycle more often), while the other two configuration variables do not. The change in maximum velocity that results from alterations in myosin step size and detachment rate are a result of differences in how long a myosin remains in its power or drag-stroke state; in motility systems the total force of the system must equal zero, which suggests that myosins with longer drag-strokes or shorter power-strokes will generate more negative force or less positive force, respectively, therefore resulting in lower system filament velocities.

Although there is limited empirical evidence describing the loaded response of synthetic isoforms with altered variables in comparison to chicken skeletal muscle myosin, there is experimental evidence describing the maximum (or unloaded) velocity *v*_*u*_ for synthetic myosins [14] with altered *δ*_*+*_ and natural myosins of varied *k*_*off*_ [33], while *k*_*on*_ alterations have no influence, which is plotted in Fig 3B. Analytically *v*_*u*_ is found using Eq 2, while the simulation requires an *a priori* assumed velocity and determination for whether the time-average force of the system equals zero. The unloaded velocity for simulated systems was found by iteratively adjusting the input *v* in increments of 100 *nm/s* from zero until 〈*d*〉 ≤ 0*nm*, and is plotted in Fig 3B in addition to the empirical results. There is strong agreement among analytical and empirical trends, suggesting that the biophysics is captured for each of the unique influences of isoform configuration inputs. The juxtaposition of these two modeling approaches is important, as the simulation determines relationships by allowing system relationships to emerge in contrast to explicit formulations from the analytical model, but both approaches result in similar predictions.

### Stochastic Ensemble Behavior

**Fig 4:**
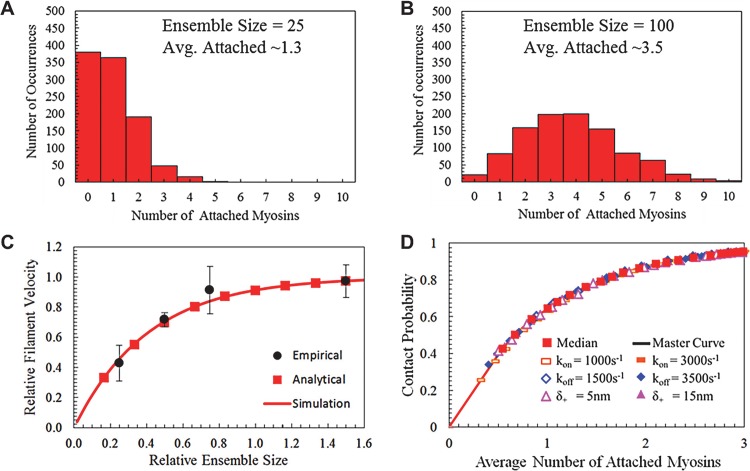
Stochasticity affects velocity and attachment among myosins and filaments for varied ensemble sizes. Histograms for simulated ensembles of (a) *N* = 25 myosins and (b) *N* = 100 myosins when *δ*_+_ = 10*nm*, *k*_*on*_ = 900*s* ^−1^, and *k*_*off*_ = 1600*s* ^−1^ as the number of attached myosins are counted for 1000 random samplings (c) The normalized unloaded filament velocity *v*_*u*_ for the isoform from “a” when considering ensemble size for analytical predictions (*N* = 60 myosins), simulation (*N* = 60 myosins), and empirical data (normalized to 100μg/mL concentration of chicken skeletal myosins added to a flowcell). (d) Analytical curve of contact probability *P*_*C*_ and average number of attached myosins *N*_*att*_ for a median isoform of *δ*_+_ = 10*nm*, *k*_*on*_ = 2000*s* ^−1^, and *k*_*off*_ = 2500*s* ^-1^ as ensemble size varies. All simulated isoforms are identical to the median except for one perturbed parameter as indicated in the key, with all outputs collapsing on a single curve. Therefore, systems have nearly identical contact probabilities for a given number of attached myosins, independent of ensemble size or isoform configuration.

The analytical and simulation models are both extendible to predicting stochastic ensemble behavior, such as determining the probability that at least one myosin in the system is attached to actin, which is necessary for ensuring the system operates with a consistent trajectory and does not dissociate [27]. The contact probability *P*_*C*_ that describes the percentage of time that at least one myosin is attached to actin is used to find an adjusted unloaded filament velocity $v_{u}^{*}=P_{c}∙v_{u}$. To determine the simulated *P*_*C*_, Monte Carlo methods were used to count the number of attached myosins during run-time. Fig 4A and 4B are histograms for ensembles of *N* = 25 myosins and *N* = 100 myosins, demonstrating that occurrences obey a Poisson distribution, and there is a much lower chance of no myosins being attached as *N* increases.

The contact probability *P*_*C*_ can then be found from the simulation by taking the proportion of measurements with zero myosins attached compared to the total number of measurements, which is comparable directly with empirical and analytical results (through using the relationship $v_{u}^{*}=P_{c}∙v_{u}$). Experimentally, data was collected using chicken skeletal muscle myosin and increasing the concentration of myosins added to a flow cell while measuring actin filament velocity, until adding more myosins to the system resulted in no further increases in motility velocity, which is indicative of the point when at least one myosin remains in contact with actin about 100% of the time [21] (Supplementary Movie 2 in S1 Text). In these experiments, a viscous liquid was added to the motility cell environment in order to reduce the chance of system dissociation when no myosins were attached, and enables the measurement of filament velocities relative to the highest possible velocity for the system (Fig 3C). This point was reached in experiments when amounts greater than 100 μg/mL of myosins were added to the motility cell. When simulation and experimental results are normalized for the case of chicken skeletal muscle myosins (*k*_*on*_ = 900*s* ^−1^, *k*_*off*_ = 1600*s* ^−1^, and *δ*_+_ = 5*nm*) as N varies, it was found that strong agreement occurs when plotted with N = 60 myosins as the ensemble size that corresponds to the maximum achievable velocity (Fig 3C), and reflects ensembles with a contact probability *P*_*C*_ of about 91%. Uncertainty in these comparisons exist because it is not possible to determine with certainty the number of myosins interacting with actin filaments empirically, to measure the average filament velocity of the system with certainty, and because there are likely fluctuations in instantaneous filament velocities that occur physically, but are not represented in the models.

Instead of precisely calculating processive lifetime, analytical approaches often rely on approximations such as the rule suggesting that if at least two myosins are attached to a filament on average, *N*_*att*_ ≥ 2, a system will remain perpetually processive. When *N*_*att*_ = 2, it is generally true that *P*_*C*_ ≈ 90% for chicken skeletal muscle myosin [19]. It is possible to compare the simulation and analytical methods when considering many isoforms and *N*, to determine whether all systems of a given *N*_*att*_ will have similar *P*_*C*_ according to *P*_*C*_ = 1 – (1 – *r*)^*N*^. The analysis is conducted by first choosing a median isoform (*k*_*on*_ = 2000*s* ^−1^, *k*_*off*_ = 2500*s* ^−1^, and *δ*_+_ = 10*nm*) and increasing the amount of myosins within the system until the average number of attached myosins is three, which results in a curve of *N*_*att*_ and *P*_*C*_ for that particular isoform (Fig 4D). The process was repeated for isoforms that have one isoform value different in comparison to the median isoform, which enabled a controlled basis of comparison to determine how each myosin isoform configuration input affected ensemble energy and processivity behavior. For instance, the curve “*k*_*on*_ = 1000*s* ^−1^” in the legend of Fig 4D indicates ensembles of different sizes for an isoform with values of *k*_*on*_ = 1000*s* ^−1^, *k*_*off*_ = 2500*s* ^−1^, and *δ*_+_ = 10*nm*. Isoforms were extrapolated one at a time for each isoform input parameter and resulted in a total of seven data sets that all followed the same curve in Fig 4D, thus indicating the basic relationship between the number of attached isoforms and probability that at least one was attached for all considered system configurations. The values of isoforms chosen for extrapolation in Fig 4D represent a set of isoforms within 2–3 times greater or smaller parameter values when compared to chicken skeletal muscle myosin, which forms a significant basis of comparison, while also being representative of known myosins with larger lever arms [36] and faster kinetics [38].

The collapsing of all isoform types on the same curve in Fig 4D occurs even though systems have isoforms of differing *r* and varied *N* to reach a particular *P*_*C*_, and is related to the number of attached motors obeying a Poisson’s distribution. Therefore, either *P*_*C*_ or *N*_*att*_ could interchangeably scale with P. Because P measurements are expected to scale exponentially with *N* as supported experimentally [25], we propose a unified expression in the form $P(N_{att})=Ae^{B∙N_{att}}$;, where *N*_*att*_ increases with *N* and *r*, and *A* and *B* are scaling constants. The exponential scaling assumption also follows from Fig 4A and 4B, because of the low chance of no myosins being attached in large ensembles. The scaling equation only holds if *A* and *B* have similar values for all isoforms, a property tested with simulations. If accurate, the equation enables the prediction P from any system based on *N*_*att*_, which is a property of the system and not of single myosins and is difficult to validate with analytical and experimental approaches.

### Processive Lifetime Simulations

**Fig 5:**
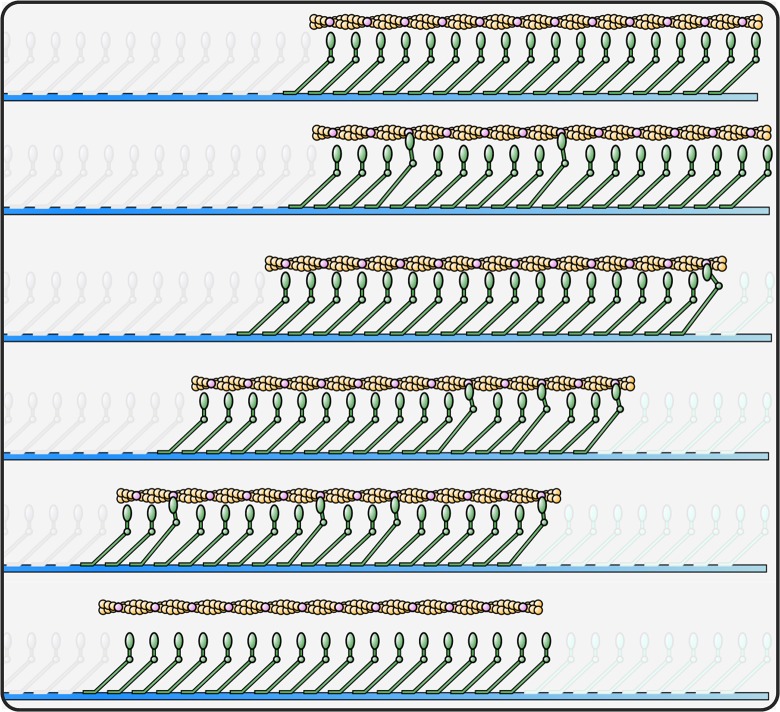
Processive myosin simulation rendering. The rendering illustrates six periods of time during the agent-based simulation of a single processive run-length event. In the first (top) frame, no myosins are attached, then myosins begin attaching and propelling the filament until a period of time greater than 1ms when no myosins are attached, which leads to systems dissociation (bottom frame). The duration of time recorded for the run length event is measured from the initial point of myosin contact with a filament until system dissociation.

To determine whether the unified expression predicts system processivity independently of individual isoform configuration, simulations must demonstrate that all isoforms have similar coefficients *A* and *B*. To determine these coefficients, the computational environment was modified to recreate processive lifetime events, with each simulation measurement reflecting the time from initial myosin-actin contact until system dissociation (Supplementary Movie 3 in S1 Text). Dissociation occurs in the simulation when no myosins are attached for 1*ms*, which represents an average duration before an actin diffuses from the myosins’ reach, based on past experiments [39]. It is possible to manipulate this duration through altering the fluid’s viscosity in the environment. When histograms for processive lifetime P were produced from simulations of two different ensemble sizes *N* (Supplementary Movie 4 in S1 Text), they followed an exponential decay that agrees with past experiments [36] (Supplementary Section 1 in S1 Text). A sample processive run recorded from the simulation environment is presented in Fig 5, and illustrates a duration of time from initial contact among myosin and actin until system dissociation occurs when no myosins are attached for a period of greater than 1 *ms*.

**Fig 6:**
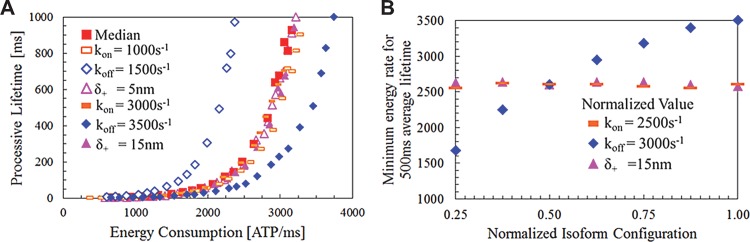
Trends among isoform variations for simulated processive lifetimes and ensemble energy usage. (a) Simulation measurements of processive lifetime P and contact probability *P*_*C*_ when ensemble size *N* varies. Isoforms include a median (red with *δ*_+_ = 10*nm*, *k*_*on*_ = 2000*s* ^−1^, and *k*_*off*_ = 2500*s* ^−1^), with other isoforms having one perturbed parameter as indicated, therefore higher contact probabilities lead to longer P, and lower detachment rates *k*_*off*_ lead to higher processive lifetimes for a given contact probability. (b) The minimum average system energy consumption *E* required for P≥500ms. Isoforms are all perturbed from a configuration where parameters are half of their normalized value; isoforms of higher *k*_*off*_ require more energy to reach the same P for a given *P*_*C*_.

An investigation of many different ensemble sizes and isoform configurations was conducted using the simulation to measure processive lifetime of varied isoform configurations (Supplementary Movie 5 in S1 Text). A significant body of data was collected, beginning with the simulation of a median isoform configuration (*k*_*on*_ = 2000*s* ^−1^, *k*_*off*_ = 2500*s* ^−1^, and *δ*_+_ = 10*nm*) with an ensemble size *N* of 10 while myosin and system behaviors were recorded from the simulation. The ensemble size of the system was increased until the average processive lifetime exceeded 1s (higher values of processive lifetime began approaching perpetually processive systems that required extensive computational effort). The process was repeated for isoforms that varied by one input variable in comparison to the median isoform (e.g. an isoform with extrapolated *k*_*on*_ = 1000*s* ^−1^ represented an isoform of *k*_*on*_ = 1000*s* ^−1^, *k*_*off*_ = 2500*s* ^−1^, and *δ*_+_ = 10*nm*). The extrapolation of one isoform variable from the median isoform enabled a controlled basis of comparison to determine how each myosin isoform configuration input affects ensemble energy and processivity behavior. Isoforms were extrapolated one at a time to produce seven curves that represented how each ensemble's contact probability *P*_*C*_ corresponded to its processive lifetime in Fig 6A.

Results in Fig 6A demonstrate an exponential increase in P that scales with system energy consumption (and therefore contact probability P_c_), as expected. However, the constants *A* and *B* are not conserved because myosins with higher detachment rates *k*_*off*_ have much lower P for a given system energy consumption. These results suggest that processive lifetime scaling for systems of different isoforms does not occur universally with contact probability, but could possibly occur through considering relationships with system energy consumption when considering that higher *k*_*off*_ leads to increasingly unstable systems with higher energy requirements $E_{req}\left(P\right)$ to reach a given P [17]. The energy requirement arises from considering the average ATPase rate of a myosin *e* = *v*/Δ and assuming a minimum number of myosins are required *N*_*req*_ to reach a given P such that $N_{req}\left(P\right)=N_{att}\left(P\right)/r$. It follows that a required system energy is $E_{req}\left(P\right)=e∙N_{att}\left(P\right)/r$, and in terms of isoform parameters is

$$E_{req}\left(P\right)=\frac{N_{att}\left(P\right)∙v}{\delta_{+}+v∙{\left(k_{off}\right)}^{-1}}$$ (6)

The relationship among myosin isoform values being predictive of the energy required for processivity may be investigated through viewing simulation results according to the energy consumed by a system on average for a given processivity. The process is initiated by considering how $E_{req}\left(P\right)$ fluctuates as each myosin parameter is altered independently. Eq 6 can be simplified to $E_{req}\left(P\right)=N_{att}\left(P\right)∙k_{off}$, suggesting that *E*_*req*_ scales with *k*_*off*_. This agrees with simulations because *N*_*req*_ remains constant for myosins of varied step sizes *δ*_+_ (as duty ratio *r* remains unchanged) while *E*_*req*_ remains static as attachment rate *k*_*on*_ varies despite *e* varying (Supplementary Section 2 in S1 Text). *E*_*req*_ was determined at P=500ms by simulating increasingly larger ensembles until reaching the first occurrence of P≥500ms from an average of 1,000 simulations for a varied set of isoforms that includes a baseline isoform with *k*_*on*_ = 1250*s* ^−1^, *k*_*off*_ = 1500*s* ^−1^, and *δ*_+_ = 7.5*nm*, and isoforms with one input parameter extrapolated from the baseline isoform values (Fig 6B). Fig 6B demonstrates that *E*_*req*_ grows with *k*_*off*_ but remains constant as other parameters vary. Therefore, Eq 6 is not a fully predictive model and the unified scaling law requires adjustment to account for the energy differences in processivity when *k*_*off*_ is altered, which suggests a modification to find an adjusted system energy E* = *E*_*sys*_ ∙ *N*_*att*_.

### Unified Scaling at the Systems Level for All Isoforms

**Fig 7:**
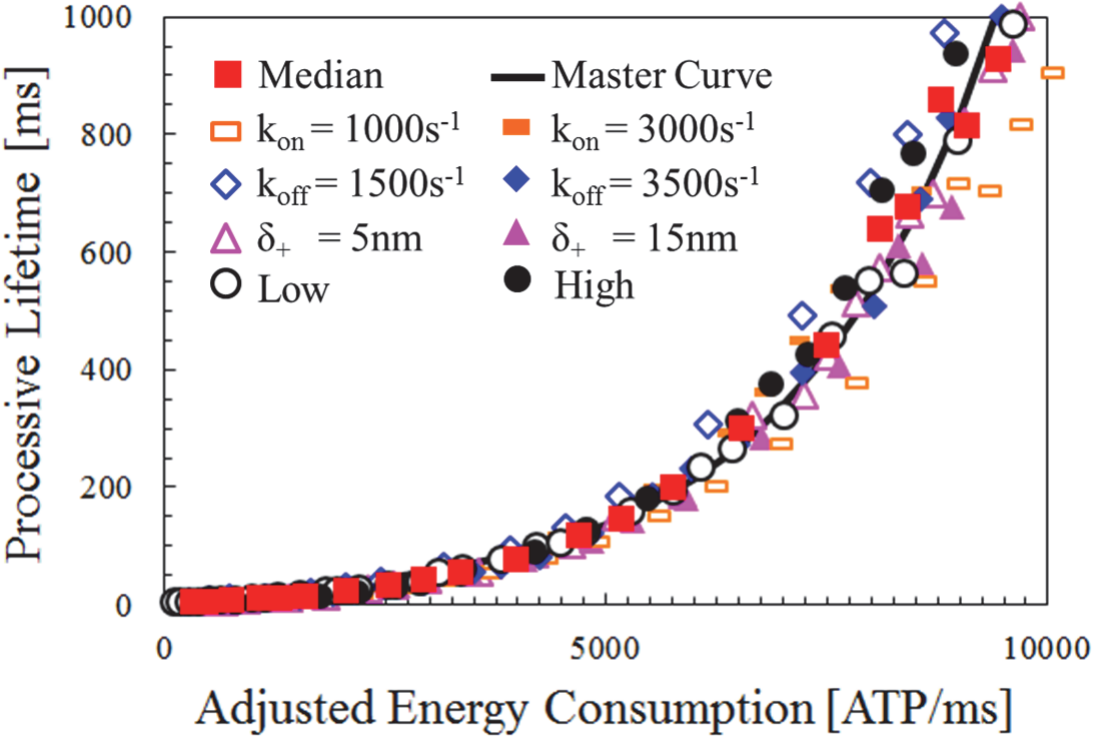
Master curve that predicts processive lifetime of ensembles composed of many different myosin isoforms. Processive lifetimes for isoforms when adjusted system energy consumption E* varies. Isoforms are identical to Fig 6A, except for additional low (*δ*_+_ = 5*nm*, *k*_*on*_ = 1000*s* ^−1^, and *k*_*off*_ = 1500*s* ^−1^) and high (*δ*_+_ = 15*nm*, *k*_*on*_ = 3000*s* ^−1^, and *k*_*off*_ = 3500*s* ^−1^) isoforms, which demonstrate the master curve holds as multiple myosin parameters are altered. The master curve analytically captures the overall response predicted through the unified expression $P\;=\;Ae^{B∙E^{*}}$, with *A* ≈ 14.5 and *B* ≈ 4.5(10^−4^). Here, processive lifetime is predictable regardless of individual myosin configuration and the master curve asymptotes are indicative of energy thresholds for perpetual processivity.

When determining E* from Fig 6B simulation results are representative of ensembles with processive lifetimes of approximately 500ms. All isoforms have nearly identical E* while having vastly different contact probabilities, thus suggesting that E* is a viable predictor of processive lifetime (Supplementary Section 3 in S1 Text). Therefore, the unified expression that fits the simulation data is possible to express as $P(E^{*})=Ae^{B∙E^{*}}$. When simulation results from Fig 6A are reconsidered with E*, there is strong agreement among all isoform types adhering to one master curve (Fig 7). Coefficients were fit to the median isoform in Fig 7, resulting in *A* ≈ 14.5 and *B* ≈ 4.5(10^−4^) and even hold as multiple parameters of isoforms are varied simultaneously (as represented by “low” and “high” isoform configurations in Fig 7).

The results demonstrate that ensembles have similar processive lifetimes as a function of adjusted energy consumption E*, regardless of which isoform is present. These results are significant, because they suggest that the properties of the myosin motility system scale independently of the myosins configured within the system, despite individual changes in myosins having unique effects on other performance metrics as considered in previous figures (Figs 3A, 3B and 6A).

## Discussion

In this study, agent-based simulation techniques were utilized to find a unified expression for describing processive myosin ensemble lifetimes P that hold as ensembles size and single myosin structures vary. Two hypotheses were tested that were informed by past experimental data, that P scales with the number of myosins in a system or the probability that at least one is attached. Simulations demonstrated that neither of these hypotheses held true. Instead, a master expression was derived that enables novel quantifications of P through consideration of how the number of attached myosins influences energy consumption. The expression was determined through simulation evidence that although P always increases with larger contact probabilities and system energy for a given isoform, systems energy modulation potentially offers a more accurate scaling law that holds for all systems regardless of isoform presence. The unified expression simplifies understanding and analysis of myosin systems, as emergent system behavior is quantifiably independent of system sub-components.

These results are significant in that they investigate critical parameters in a complex system and find fundamental laws based on a resulting model with stochastic components. The model makes several simplifying assumptions that could be further investigated as potential indicators of universal system behaviors. For instance, myosins have many different mechanochemical states and under certain circumstances, such as operating at very low velocities or high forces, other biophysical mechanisms may alter aspects of the three state cycle significantly. Additionally, the model makes a simplifying assumption that the system operates at a constant velocity and force regardless of how many myosins are attached to the filament; it is expected that since myosins may not contribute equal force to the filament over their entire cycle, the filament velocity would fluctuate with low myosin counts. These considerations suggest that the results of the unified master equation are applicable for the particular set of isoforms and assumptions modeled in this study, and further considerations of laws may be required to determine their applicability when the system enters differing regimes of emergent behavior [40] or operates under largely different conditions.

Loading on myosin systems may also influence their processive functioning, although it is difficult to measure motility assay performance with known loads [21]. These situations were not investigated in the study as there is no validation for varied myosin isoforms experimentally. In the analytical and agent-based models of myosin, additional loads in the system would only alter the processive lifetimes of systems through reducing the filament velocity, since myosin cycles are dictated in the model entirely by the speed of the gliding filament. The reduction in filament speed would occur because myosins are required to balance forces among themselves and the external load. However, it is possible that empirical studies would show that processive myosin systems behave quite differently under load, because single myosins are no longer able to propel the filament at all, therefore possibly promoting a more stochastic instantaneous velocity.

The method for finding a unified scaling expression using an agent-based simulation approach is extendible to describing emergent laws in a variety of similar complex biological systems. The derivation of such laws is particularly important because they enable a simplification in the analysis of systems with many different parameters, and could form heuristics for engineers to follow when designing nanotechnologies. Through finding universal relationships in how the configuration of a system affects its behaviors, it enables researchers to concentrate on other trade-offs present in the system, such as choosing among isoforms on the basis how fast they propel filaments under conditions that control for desired processive lifetimes across systems. Such considerations in simplifying the modeling and variables are crucial, both in promoting understanding from a scientific perspective and enabling effective development of bionanotechnologies.

## Supporting Information

S1 Text Contains descriptions of Supplementary Movies, Supplementary Section 1 (Histogram of simulated processivity events), Supplementary Section 2 (Influences of Varied Isoforms on Processivity), and Supplementary Section 3 (Determination of a Unified Scaling Equation). (DOCX)

S1 Dataset Simulation and empirical data. (XLSX)
