# Supplementary material for: Emergent Systems Energy Laws for Predicting Myosin Ensemble Processivity
Source: PLoS Comput Biol. 2015 Apr 17;11(4):e1004177. doi: 10.1371/journal.pcbi.1004177 (PMC4401713; doi:10.1371/journal.pcbi.1004177)
Supplement: S1 Text — (DOCX) [file pcbi.1004177.s001.docx]

Supplementary Text

**Supplementary Movies**

There are five supplementary movies available at the following links:

SM1: Agent-based molecular simulation rendering: http://youtu.be/ImHgcoru7Sg

SM2: Flow cell microscopy data: http://youtu.be/-L2F3f_sZKM

SM3: Processive run-length simulations: http://youtu.be/grStiSNWUEw

SM4: Processivity for different ensemble sizes: http://youtu.be/HPutK_oTG4M

SM5: Processivity for different lever arm lengths: http://youtu.be/0NuHZTDo5Zc

**S1. Histogram of Simulated Processivity Events**

Ensembles of chicken skeletal muscle myosins ($k_{on}=900s^{-1}$, $k_{off}=1600s^{-1}$, and $\delta_{+}=5nm$) were modeled in the agent-based environment with varied ensemble sizes to determine the distribution of processive lifetime $\mathcal{P}$ durations over the course of a large number of measurements. Each processive lifetime event is a measurement from the first contact between myosin and actin in a system, until system dissociation occurs. In the simulation environment, system dissociation occurs if no myosins in the system are attached to actin for a duration of 3ms. Because myosins attach and detach from actin based on stochastic rules, it is expected that that processive run-length will obey a poisson’s distribution. In order to compare the differing distributions and average lifetimes for small and large ensembles, 3000 processive lifetime simulations were conducted for varied ensemble sizes. Histograms were generated to demonstrate the frequency of processive-lifetime measurements for systems with 40 myosins (Fig. S1a) and 60 myosins (Fig. S1b).

**
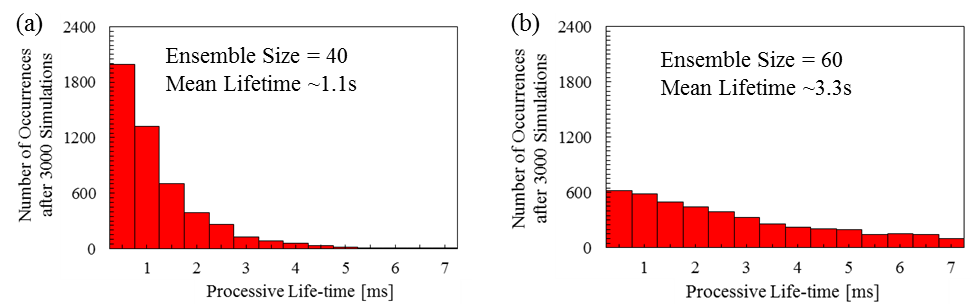
**

**Fig. S1: Processive lifetime simulation measurements.** The processive lifetime of an ensemble of chicken skeletal muscle myosins ($k_{on}=900s^{-1}$, $k_{off}=1600s^{-1}$, and $\delta_{+}=5nm$) was simulated 3000 times and plotted as a histogram for ensemble sizes of (a) 40 and (b) 60. Mean lifetime represents the average measured value for each plot.

The results show that there is an exponential decay in processive lifetimes at low ensemble sizes, with many more occurrences of short life-times. As more myosins are added, it does not completely remove the chance of a system dissociating with a low processive lifetime, but there becomes a much higher probability of long processive lifetimes.

**S2. Influences of Varied Isoforms on Processivity**

To understand the effects of each isoform configuration variable on processive lifetime $\mathcal{P}$ and the use of energy at the individual and ensemble level systems of varied ensemble sizes and isoforms were simulated as the energy expenditure of myosins were tracked for each processive lifetime event. A median isoform configuration ($k_{on}=2000s^{-1}$, $k_{off}=2000s^{-1}$, and $\delta_{+}=10nm$) was simulated with an ensemble size 𝑁of 10, and 1000 processive lifetime simulations were conducted. The size of the system was then increased until average processive time exceeded 1s, and 1000 processive lifetime simulations were conducted for each ensemble size. Then, the process was repeated for isoforms that varied by one variable in comparison to the median isoform, which enables a controlled basis of comparison to determine how each myosin isoform configuration input affects ensemble energy and processivity behavior. Isoforms were extrapolated one at a time below and above that value to produce seven curves that represent ensembles of different myosins varied as a function of ensemble size $N$ (Fig. S2a) or system energy $E$ (Fig. S2b).


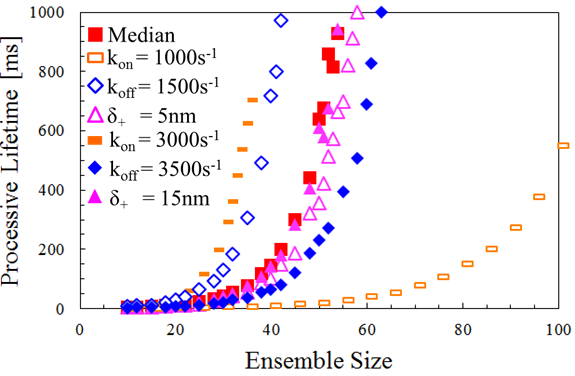


**Fig. S2: Processive lifetime simulations of varied isoforms for ensemble size increases.** The median isoform has input values of $k_{on}=2000s^{-1}$, $k_{off}=2500s^{-1}$, and $\delta_{+}=10nm$ while all additional isoforms are identified by the input value that differentiates them from the median isoform. For instance, the isoform in the legend identified as “$k_{on}=1000s^{-1}$” represents an isoform with values $k_{on}=1000s^{-1}$, $k_{off}=2500s^{-1}$, and $\delta_{+}=10nm$. Resulting simulations produced curves for each isoform for processive lifetime as a function of ensemble size.

The results show that as myosin step size $\delta_{+}$ is varied, all isoforms have nearly equivalent processive lifetimes as a function ensemble size. This phenomenon occurs because at an unloaded velocity $v_{u}$ the filament is typically moving fast enough that the power-stroke phase of a myosin’s cycle becomes negligible as $\lim_{v\to\infty} r(v)\propto\frac{k_{on}}{k_{off}}$. Therefore, the step size $\delta_{+}$ has no influence on duty ratio $r$ and the contact probability$P_{c}=1-{(1-r)}^{N}$ only varies as a function of $N$ if the step size of myosins is varied while other myosin input variables are held constant. However, isoforms of varied detachment rates $k_{off}$ and attachment rates $k_{on}$ do vary in processivity as a function of ensemble size $N$ as demonstrated by the figure.

In the case of attachment rate being the manipulated variable as other myosin inputs remain constant the system is operating with a different amount of energy consumption for a given ensemble size, because myosins with greater attachment rates cycle more frequency, therefore consuming more energy $e$ (each myosin uses one ATP per cycle) at a faster rate (Fig. S3a). When $E$ is found through aggregating $e$ of all myosins in a system in the simulation or analytically by $e=v/\Delta$. It is found that all myosins with altered $k_{on}$ have nearly equivalent $\mathcal{P}$ as a function of $E$ when investigating with the agent-based simulation (Fig. S3b).


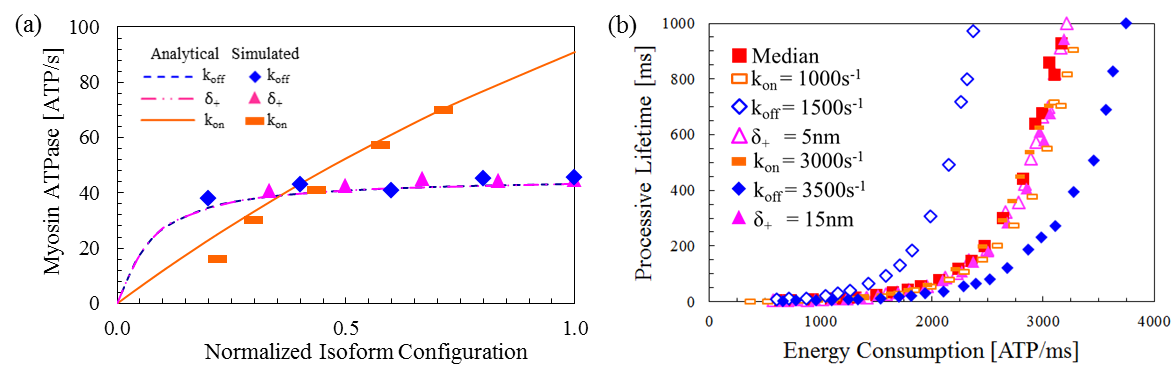


**Fig. S3: ATPase of varied myosin isoforms and energy expenditure of ensembles.** (a) Single myosin ATPase as a function of the same normalized isoform configurations, where the maximum value of a perturbed parameter for each isoform has a value of 1, where the median isoform is identical to that of Fig. S2. (b) The processive lifetime of ensembles from Fig. S2 as a function of Energy Consumption.

Through examining the analytical equations and the assumption that ${\mathcal{P(}N}_{att}) \propto{\mathcal{P(}P}_{c})$ (Fig. 4d), it is can be demonstrated why all myosins of altered attachment rates produce systems where processive lifetime scales with system energy consumption: Because $N_{att}$ grows proportionally with $r$, in order to retain the same $N_{att}$ that corresponds with a given $P_{c}$, $N$ must change. Support for the energy modulation hypothesis comes from considering $E_{req}\left( \mathcal{P} \right)=e_{myo}\cdot N(\mathcal{P)/}r$ remains static as a function of $k_{on}$. Therefore both of the proposed hypotheses, that $\mathcal{P}$ scales with $P_{c}$ or $E$ hold for myosins of varied $\delta_{+}$ and $k_{on}$. It is only through examining Figures 6a and S3b that simulation results suggest a contradiction among the two hypotheses for myosins of altered $k_{off}$, which do not have a unified scaling as a function of ensemble size or ensemble energy consumption.

**S3. Determination of a Unified Scaling Equation**

A scaling expression that is not dependent on detachment rate $k_{off}$ can be found by adding terms to the system energy term until it is unaffected by $k_{off}$, to maintain its consistency for the other myosin input variables. Through simplification of equations, it is found that $E_{req}\left( \mathcal{P} \right)=E(\mathcal{P)/}k_{off}$, which can be manipulated to form an expression that scales independently of $k_{off}$ and all other configuration variables through proposal of an adjusted system energy consumption parameter $E^{*}$, defined as $E^{*}=E_{sys}\cdot N_{att}$. To test this equation, the data from Fig. S2 for each isoform that corresponds to a processive lifetime value of about $\mathcal{P}=500ms$ is plotted as a function of outputs that could act as predictors for a unified equation as a function of contact probability (Fig. S4a) and adjusted system energy consumption (Fig. S4b). Additionally in Figure S4, “Low” and “High” isoform ensembles are representative of isoforms where all parameters are perturbed in comparison to the median (e.g. the “Low” isoform has values of $k_{on}=1000s^{-1}$, $k_{off}=1500s^{-1}$, and $\delta_{+}=5nm$), which was conducted because a unified scaling law should account for simultaneous manipulations of multiple myosin input parameters.


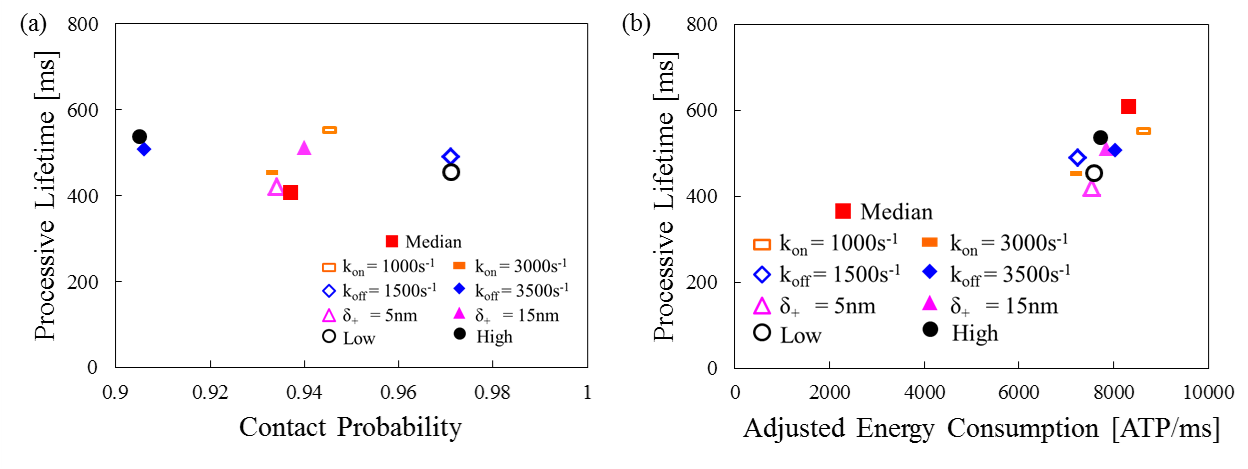


**Fig. S4: Scaling of isoforms with ensemble configured to have processive lifetimes of about 500ms.** Isoforms from Fig. S2 are plotted for processive lifetime as a function of (a) contact probability and (b) adjusted system energy consumption.

In Figure S4a, it is found that contact probability (and therefore system energy) does not act as a strong predictor of processive lifetime. However, Figure S4b demonstrates that the processive lifetime has a positive correlation with adjusted energy consumption when all isoforms are considered together. The slight differences in adjusted energy consumption and processive lifetime of the isoforms is a product of not being able to solve for $\mathcal{P}=500ms$ for each isoform because it is not possible to run the simulation with fractional number of myosins (i.e. an ensemble size of 57 undershoots the processive lifetime target while an ensemble size of 58 leads to processive lifetimes that overshoot the target value). These results suggested that adjusted system energy consumption could be utilized as a unified scaling factor for all isoforms if more data points are considered for different processive lifetimes, which was demonstrated in Figure 7 of the primary manuscript.
